# Comparative Studies of 5S rDNA Profiles and Cyt *b* Sequences in two *Onychostoma* Species (Cyprinidae)

**DOI:** 10.3390/ijms161226193

**Published:** 2015-12-11

**Authors:** Chiao-Chuan Han, Tsair-Bor Yen, Nian-Cih Chen, Mei-Chen Tseng

**Affiliations:** 1 National Museum of Marine Biology and Aquarium, Pingtung 944, Taiwan; hancc@nmmba.gov.tw; 2 Graduate Institute of Marine Biology, National Dong Hwa University, Hualien 974, Taiwan; 3 Department of Tropical Agriculture and International Cooperation, National Pingtung University of Science and Technology, Pingtung 912, Taiwan; tbyen@mail.npust.edu.tw; 4 Institute of Aquaculture, National Pingtung University of Science and Technology, Pingtung 912, Taiwan; niancih0701@gmail.com

**Keywords:** adaptation, cyprinid, cytogenetic, genetic distance, Taiwan

## Abstract

*Onychostoma barbatulum* and *O. alticorpus*, two primarily freshwater cyprinid fish, have similar morphological characters and partially overlapping ecological habitats. In order to explore the genetic differences between these two species, chromosomal characteristics and genetic variations were examined by fluorescence *in situ* hybridization (FISH) of 5S rDNA and cytochrome (Cyt) *b* gene analysis. Ten specimens of *O. barbatulum* and *O. alticorpus* were collected from the Nanzihsian Stream in southern Taiwan. FISH revealed that the 5S rDNA loci of *O. barbatulum* and *O. alticorpus* were found at a pericentromeric and subtelomeric position, respectively, in a pair of submetacentric chromosomes. Cyt *b* genes were amplified and sequenced from five individuals of each species. Intraspecific genetic distances ranged from 0.001–0.004 in *O. barbatulum* and from 0.001–0.006 in *O. alticorpus*. Genetic distances between these two species ranged from 0.132–0.142. The phylogenetic tree showed these two species are not sister species. In conclusion, FISH cytogenetic information and Cyt *b* gene analyses indicated that these two species have significantly different genetic characteristics; nevertheless, their morphological similarities may be due to environmental adaptation.

## 1. Introduction

**Figure 1:**
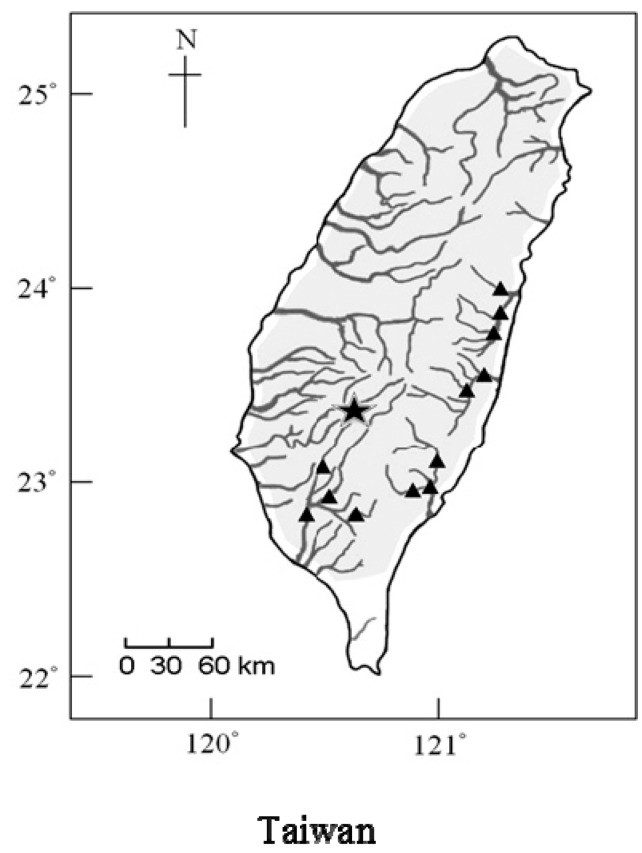
Distribution of *Onychostoma barbatulum* (gray area) and *O. alticorpus* (black triangles). Asterisk indicates the sampling location.

The genus *Onychostoma* contains about 20 species distributed widely in East Asia, and some of them are economically important. Both *O. barbatulum* (Pellegrin, 1908) and *O. alticorpus* (Oshima, 1920) reside on Taiwan Island and are cyprinid fish that have similar external morphological traits and almost identical body shapes before reaching a length of about 7 cm. Consequently, it is difficult to distinguish young individuals of these two species based on external traits. Upon reaching adulthood, their body shapes gradually become distinctive due to *O. alticorpus* expressing a greater body depth and a more obvious fusiform shape than *O. barbatulum*. In addition, they share partially sympatric habitats. Most individuals of *O. barbatulum* and *O. alticorpus* dwell along the stream bottom, feeding on algae and aquatic insects, but they prefer different micro-environments and have different distributions. *O. barbatulum* resides in upper stream but migrates to midstream or downstream for winter feeding [1]. It is found in all of Taiwan’s rivers except the smaller streams of the southernmost Hengchun [2]. The Taiwan-endemic *O. alticorpus* resides in the midstream and downstream year-round, and has a narrower south-to-east distribution range that includes the Kaoping, the Hsiukuluan, the Beinan, and the Taimali Rivers (Figure 1) [3,4]. However, previous studies considered the two species to have seasonal sympatric habitats.

The development of cytogenetics with modern molecular techniques that has resulted in a better understanding in genome structure and organization provides a useful tool to study species evolution and efficiently characterize species and populations [5,6]. The cytogenetics of *Onychostoma* is currently not fully understood and only a few basic studies have been published simply comprising chromosome number and fundamental arm number [7,8,9]. The highly limited cytogenetic techniques applied to fish chromosomes have been greatly thrust by the convenience of fluorescence *in situ* hybridization (FISH) and FISH-derived procedures [10]. Repetitive DNAs represent a large fraction of vertebrate genomes and are the most frequently used probes to study the organization of fish genomes [11], particularly the multigene family of ribosomal DNAs (rDNAs) [12,13]. It is usually found at one or several different chromosomal loci with a tandem repeated arrangement in higher eukaryotes [14]. Molecular tools, especially the cytochrome *b* (Cyt *b*) gene, are routinely used in studies of species identification and systematics [15,16].

The aim of the present study was to explore the genetic differences and evolution of *O. barbatulum* and *O. alticorpus* by examining their 5S rDNA profiles using FISH and Cyt *b* gene analysis.

## 2. Results and Discussion

**Figure 2:**
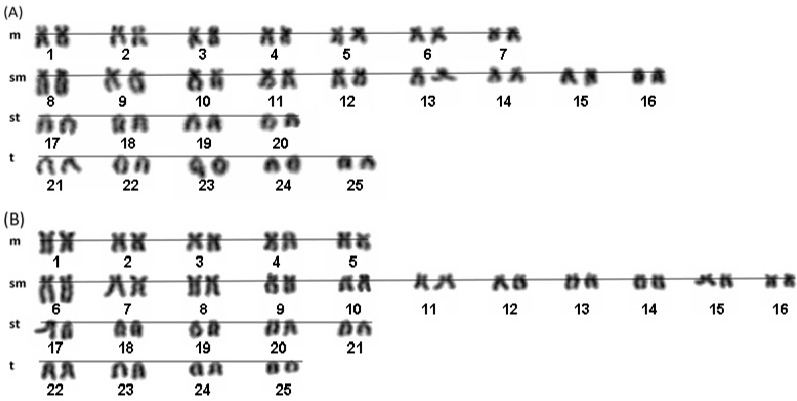
Karyotypes of *O. alticorpus* (**A**) and *O*. *barbatulum* (**B**) females after Giemsa staining. Both species show 2*n* = 50; m = metacentric, sm = submetacentric, st = subtelocentric, t = telocentric.

Cytogenetic studies by Giemsa staining of *Onychostoma barbatulum* and *O. alticorpus* indicated that the diploid number for both species was 2*n* = 50 (Figure 2). The Cyprinidae, which contains more than 2400 species, is the richest family of fish with very divergent chromosome patterns [9,17,18]. The diploid number of 2*n* = 50 should be a synapomorphic character of cyprinids. In this study, *O. barbatulum* and *O. alticorpus* also have the ancestral and presumably primitive characteristic of 2*n* = 50. *O. barbatulum* and *O. alticorpus* had previously been classified *Varicorhinus* [1] based on external traits of the mouth. However, *V. nelspruitensis* Gilchrist and Thompson, 1911, *V. beso* Rüppell, 1836 and *V. capoeta* *sevangi* (De Filippi, 1865) are known for a much larger chromosome number (2*n* = 150) [19,20,21,22] than *O. barbatulum* and *O. alticorpus*.

In our study, the karyotypic formulae of female *O. barbatulum* and *O. alticorpus* were 10 m + 22 sm + 10 st + 8 t with fundamental arm numbers (FN) = 82 and 14 m + 18 sm + 8 st + 10 t with FN = 82, respectively. The previous studies also reported that the karyotypic formulae of *O. simum* (Sauvage and Dabry de Thiersant, 1874) and *O. elongatum* (Pellegrin & Chevey, 1934) were 10 m + 16 sm + 16 st + 8 t and 12 m + 12 sm + 14 st + 12 t with FN = 76 and 74, respectively [8,9]. Most of their chromosomes were metacentric (m) and submetacentric (sm), which are the conservative characteristics of *Onychostoma.*

**Figure 3:**
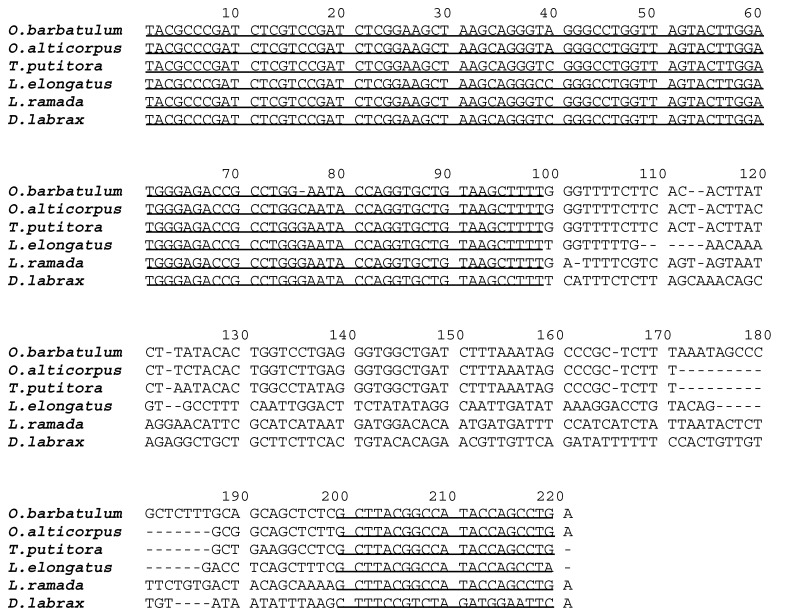
Aligned 5S rDNA sequences subcloned from *Onychostoma barbatulum* and *O. alticorpus* and four reference sequences obtained from NCBI GenBank (with accession numbers): *Tor putitora* (EU621853), *Leporinus elongatus* (AF284729), *Liza ramada* (AM706452), and *Dicentrarchus labrax* (HM014367). The first 99 bp and the last 21 bp, corresponding to the 5S rDNA transcribed sequences, are underlined.

The 5S rDNA sequences were subcloned from *O. barbatulum* and *O. alticorpus*, sequencing a total of 216 (G + C ratio = 51.9%) and 202 (G + C ratio = 52.9%) nucleotides, respectively. Their sequences were aligned to *Tor putitora* (Hamilton, 1822), *Leporinus elongatus* Valenciennes, 1850, *Liza ramada* (Risso, 1827), and *Dicentrarchus labrax* (Linnaeus, 1758), which were selected randomly from the NCBI GenBank (Available online: http://www.ncbi.nlm.nih.gov/genbank/) and have 5S rDNA sequences of high similarity (75%) across orders. In brief, it is known that the 120 bp corresponding to the transcribed region is highly conserved among distant species, while the non-transcribed regions (NTRs) are highly variable (Figure 3).

**Figure 4:**
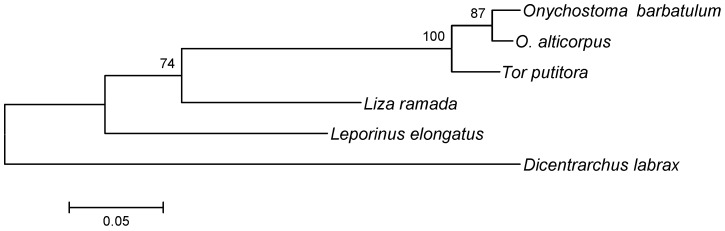
Neighbor-joining tree reconstructed based on 5S rDNA sequences from two *Onychostoma* species and four reference sequences from NCBI GenBank: *Tor putitora*, *Leporinus elongatus*, *Liza ramada*, and *Dicentrarchus labrax*. The scale bar indicates the nucleotide diversity between sequences. The bootstrapping value of each branch is shown in front of the node.

All these sequences were used to reconstruct the genealogic tree of 5S rDNA. The interspecific Kimura-2-parameter (K-2-P) genetic distance of 5S rDNA sequences between *O. barbatulum* and *O. alticorpus* (0.026) was significantly smaller than those (0.052–0.572) of *Onychostoma* to other referenced sequences in this study. A neighbor-joining analysis with an 87% bootstrapping value showed that *O. barbatulum* and *O. alticorpus* had a close relationship (Figure 4).

**Figure 5:**
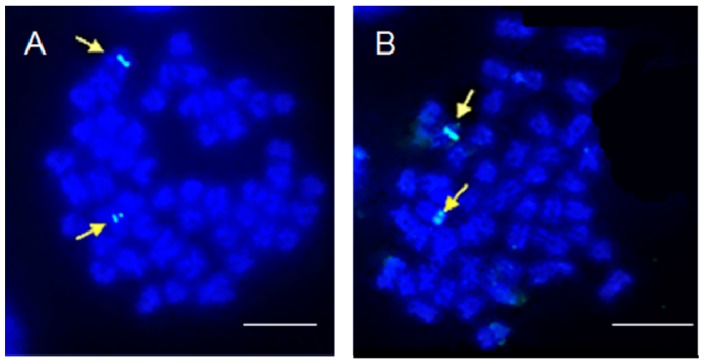
Mitotic metaphase plates of (**A**) *Onychostoma barbatulum* and (**B**) *O. alticorpus* chromosomes after fluorescence *in situ* hybridization (FISH) with 5S rDNA probes. Arrows indicate the locations of 5S rDNA loci (light blue). Bars equal 5 μm.

Two different 5S rDNA probes were prepared separately and individually hybridized with the chromosomes of these two species by FISH, both representing a single pair of signals in each species. It is noteworthy that the 5S rDNA locus of *O. barbatulum* was found at a pericentromeric region and that of *O. alticorpus* was found on the subtelomeric position of a pair of submetacentric chromosomes (Figure 5). One pair of 5S rDNA-bearing chromosomes has been presented as the plesiomorphic character state in fishes [23]. Nevertheless, the occurrence of multiple 5S rDNA sites had also been found in a few species, such as *Acheilognathus tabira* and *Cyprinus carpio* [24,25].

**Figure 6:**
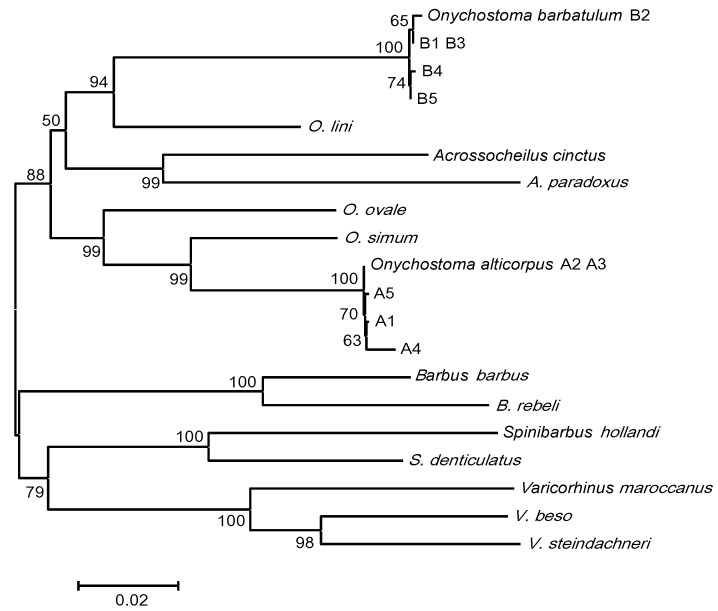
The neighbor-joining evolutionary tree reconstructed based on Cyt *b* gene sequences from five *Onychostoma barbatulum* (B1–B5) and five *O. alticorpus* (A1–A5) from Taiwan and 12 reference sequences from NCBI GenBank (with accession numbers): *Acrossocheilus cinctus* (JX066772), *A. paradoxus* (HQ443699), *Onychostoma lini* (JQ343982), *O. ovale* (JX074246), *O. simum* (HM536801), *Barbaus barbus* (KC465927), *B. rebeli* (AF090791), *Spinibarbus hollandi* (NC026129), *S. denticulatus* (NC021616), *Varicorhinus maroccanus* (AF287457), *V. beso* (JQ716388), and *V. steindachneri* (AF180865). The scale bar indicates the nucleotide diversity between sequences. The bootstrapping value of each branch is shown in front of the node.

The total length of the Cyt *b* gene was 1141 bp among 10 cloned sequences of each of the five specimens of *O. barbatulum* and *O. alticorpus* as well as in all the reference sequences of the cyprinidae species *O. ovale* Pellegrin and Chevey, 1936, *O. lini* (Wu, 1939), *O. simum*, *Acrossocheilus cinctus* (Lin, 1931), *A. paradoxus* (Günther, 1868), *Varicorhinus maroccanus* (Günther, 1902), *V. beso*, *V. steindachneri* Boulenger, 1910, *Barbus barbus* (Linnaeus, 1758), *B. rebeli* Koller, 1926, *Spinibarbus* *hollandi* Oshima, 1919, and *S. denticulatus* (Oshima, 1926). The percentages of nucleotide composition did not differ significantly among the five *Onychostoma* species. Altogether, 234 polymorphic sites encompassing 82 singleton variable sites and 152 parsimony informative sites occurred within 11 different sequences of five *Onychostoma* species. Four different haplotypes were found in both *O. barbatulum* and *O. alticorpus* and their intraspecific nucleotide diversity (Pi) values were 0.0019 and 0.0035, respectively. Intraspecific Kimura-2-parameter genetic distances among different haplotypes ranged from 0.001 to 0.004 for *O. barbatulum* and from 0.001 to 0.006 for *O. alticorpus*. The interspecific distances of *Onychostoma* samples ranged from 0.064 (*O. alticorpus* *vs.* *O. simum*) to 0.142 (*O. alticorpus* *vs.* *O. barbatulum*). Cyt *b* genes of *O. barbatulum* and *O. alticorpus* have very different nucleotide compositions. Numbers of different nucleotides between these two species ranged from 135 (A2/A3 *vs.* B5) to 144 (A4 *vs.* B2) (Table 1). A neighbor-joining analysis with 100% bootstrap support showed that specimens of *O. barbatulum* and *O. alticorpus* were on two different monophyletic branches of the phylogenetic tree (Figure 6), suggesting that they are genetically different. *O. barbatulum* and *O. alticorpus* had both been classified under *Varicorhinus* [1,3] based on their mouth shape. Therefore, their systematics still remains open to question. In this study, the intergeneric genetic distances between *Onychostoma* and *Varicorhinus* spp. ranged from 0.159 (*O. alticorpus vs. V. maroccanus*) to 0.192 (*O. barbatulum* *vs.* *V. maroccanus*) which is higher than the intrageneric distance of *Onychostoma* or *Varicorhinus* (Table 1).

*O. barbatulum* and *O. alticorpus* are the only two *Onychostoma* species presently residing in Taiwan streams. However, these two *Onychostoma* species are not sister species. In this study, the phylogeny reconstructed using the *Cyt b* gene sequence assigned *O. barbatulum* and *O. alticorpus* to different clades, not sharing a recent common ancestor. *O. barbatulum* and *O. lini* have a closer evolutionary relationship with *Acrossocheilus* sp. than with the other *Onychostoma* sp., suggesting that the *Onychostoma* genus is not monophyletic; nevertheless, *O. barbatulum* and *O. alticorpus* have similar juvenile body shapes and external morphological characteristics, which are the result of environmental adaptation.

**Table 1:** Nucleotide numbers, interspecific and intraspecific Kimura-2-parameter genetic distances (gray area) estimated from 10 cytochrome *b* gene sequences and four reference sequences. Values of genetic distances are decimals, while different nucleotide numbers are integers. Specimens A1–A5 are *Onychostoma alticorpus* and B1–B5 are *O*. *barbatulum.* Reference sequences from NCBI GenBank (with accession numbers) included Osi, *O. simum* (HM536801); Oov, *O. ovale* (JX074246); Vma, *Varicorhinus maroccanus* (AF287457); Vbe, *V. beso* (JQ716388).

	A1	A2 and A3	A4	A5	B2	B1 and B3	B4	B5	Osi	Oov	Vma	Vbe
**A1**		0.001	0.006	0.002	0.136	0.134	0.134	0.133	0.065	0.097	0.161	0.168
**A2 and A3**	1		0.005	0.001	0.135	0.133	0.133	0.132	0.064	0.096	0.160	0.167
**A4**	7	6		0.006	0.142	0.140	0.140	0.138	0.070	0.102	0.167	0.174
**A5**	2	1	7		0.136	0.134	0.134	0.133	0.065	0.097	0.159	0.168
**B2**	139	138	144	139		0.002	0.004	0.003	0.129	0.130	0.192	0.178
**B1 and B3**	137	136	142	137	2		0.002	0.001	0.127	0.128	0.191	0.178
**B4**	137	136	142	137	4	2		0.001	0.127	0.128	0.191	0.179
**B5**	136	135	141	136	3	1	1		0.125	0.127	0.190	0.178
**Osi**	70	69	75	70	132	130	130	129		0.095	0.166	0.169
**Oov**	102	101	107	102	133	131	131	130	100		0.164	0.172
**Vma**	162	161	167	160	188	187	187	186	166	164		0.105
**Vbe**	168	167	173	168	177	177	178	177	169	171	109	

## 3. Experimental Section

### 3.1. Fish Collection

Each of the five specimens of *O. barbatulum* and *O. alticorpus* were obtained from Nanzihsian Stream, a tributary of the Kaoping River in southwestern Taiwan (Figure 1). Live specimens were transported to the laboratory and maintained in fresh water in 500 L aquaria according to the *Guide for the care and use of laboratory animals* [26] until being sacrificed.

### 3.2. Chromosome Preparations and Staining

Before the experiment, fish were anesthetized using 140 μg/mL tricaine methanesulphonate (MS222, Sigma-Aldrich, St. Louis, MO, USA). Mitotic chromosomes were prepared from the cephalic kidney of each specimen. Briefly, cells were cultured in Minimum Essential Medium (MEM) with 15% fetal bovine serum (GIBCO, New York, NY, USA) and mitotic inhibitor (0.0001% Colchicine) using a Rota-mixer (Firstek Scientific Co., Taipei, Taiwan) at 100 rpm and room temperature for 2 h. A hypotonic solution (0.075 M KCl) was added to the cells for 30 min at room temperature and then centrifuged (3000 rpm, 5 min). Supernatant was decanted. Cells were fixed in a methanol-acetic acid solution at room temperature for 15 min followed by centrifugation (3000 rpm, 5 min), and supernatant was decanted. The fixation process was repeated thrice. One to two drops of cell suspension were dripped onto hot slides from a height of 10–30 cm and stained in 5% Giemsa solution for 10 min. Ten metaphase plates for each species were observed and photographed under a Leica DM2500 light microscope (Leica Biosystems, Wetzlar, Germany) with a BASLER charge-coupled device (CCD) scA1400-17gmASI camera (Basler AG, Ahrensburg, Germany). Metaphase displaying distinct chromosome morphology was used for preparing karyograms. Chromosome morphology was defined according to Levan *et al.* [27]. Chromosome numbers of species were counted and manually classified with BandView karyotyping software (ASI, Migdal Haemek, Israel).

### 3.3. 5S rDNA Purification, Subcloning and Analysis

DNA was extracted from 5 to 10 mg of muscle tissue of one specimen of both *O. barbatulum* and *O. alticorpus* using a Puregene Core kit A (Qiagen Sciences, Germantown, MD, USA). The 5S rDNA of the two species was amplified by a polymerase chain reaction (PCR) using 5S forward 5′-TACGCCCGATCTCGTCCGATC-3′ and reverse 5′-CAGGCTGGTATGGCCGTAAGC-3′ primers [23]. Amplification was performed in a Px2 Thermal Cycler (Thermo Fisher Scientific, Waltham, MA, USA). The reaction solution consisted of approximately 5 ng genomic DNA, 50 pmol each of the forward and reverse primers, 25 mM dNTP, 0.05–0.1 mM MgCl_2_, 10× buffer, and 5 U *Taq* polymerase (Takara Shuzo, Shiga, Japan) brought up to 125 μL with sterile water. The PCR program included one cycle of 4 min at 94 °C, 35 cycles of 30 s at 94 °C, 30 s at 62 °C, and 1 min at 72 °C, followed by a single further extension of 5 min at 72 °C. We evaluated 10 μL of this product on a 1.0% agarose gel to check the PCR success and confirm product sizes. The remaining PCR products were run on 1.0% agarose gels and purified using a DNA Clean/Extraction kit (GeneMark, Taichung, Taiwan). Purified DNA was subcloned into pGEM-T easy vector (Promega, Madison, WI, USA) and transformed into the *Escherichia coli* JM109 strain. Plasmid DNA was isolated using a mini plasmid kit (Geneaid, Taichung, Taiwan). Two clones from *O. barbatulum* and *O. alticorpus* were sequenced on an Applied Biosystems (ABI, Foster City, CA, USA) automated DNA sequencer ABI3730x1 using a Bigdye sequencing kit (Perkin-Elmer, Wellesley, MA, USA). T7 and SP6 primers were used in the sequencing reaction and the PCR cycle parameters for sequencing were 35 cycles of 30 s at 95 °C, 30 s at 50 °C, and 1 min at 72 °C. The genealogic analysis of 5S rDNA sequences depended on the neighbour-joining (NJ) method [28] and the significance of the clusters was assessed using bootstrap analysis with 1000 replications. The phylogeny analyses were carried out using MEGA software version 4.0 [29]. Several reference sequences, *Tor putitora* (EU621853), *Leporinus elongatus* (AF284729), *Liza ramada* (AM706452), and *Dicentrarchus labrax* (HM014367), from NCBI were added to this analysis.

### 3.4. Fluorescence in Situ Hybridization of 5S rDNA Probe

Labeled 5S rDNA probes of the two species were generated using a PCR fluorescein labeling mix kit (Roche, Mannheim, Germany). The reaction solution contained 10× buffer, 4 mM MgCl_2_, 200 μM dNTP, 1 μM 5S rDNA primers [23], 50 ng plasmid DNA, and 2 U *Taq* polymerase, and was brought up to 100 μL with sterile water. Fluorescein-labeled probes were purified by ethanol precipitation. Chromosomes were prepared with 100 μg/mL RNase A in 2× SSC buffer at 37 °C for 1 h, and washed thrice in 2× SSC for 5 min. Slides were then quickly immersed in a cold series of ethanol solutions (70%, 95% and 100%) for dehydrating the chromatin. After air-drying, chromosome spreads were done by exposing chromosomes to 0.005% pepsin (Roche, Mannheim, Germany) in 10 mM HCl at 37 °C for 10 min to remove residual proteins, and then washed in phosphate-buffered saline (PBS). Slides were then quickly dehydrated through a cold ethanol series and air-dried. Chromosomes were denatured at 80 °C for 5 min in hybridization buffer (2× SSC, 10% dextran sulfate, and 50% deionized formamide). All slides were placed on ice for 3–5 min prior to the addition of 35 μL hybridization buffer containing 50 ng labeling probe. Hybridization occurred at 37 °C for 12–16 h. Post-hybridization washes were carried out at 42 °C for 15 min in 1× SSC with 50% deionized formamide, followed by 0.1× SSC at 60 °C for 5 min thrice, and then rinsed thrice in PBS buffer with 0.2% Tween 20 at 37 °C for 5 min. Fluorescent signals were amplified using a rabbit anti-FITC antibody (Invitrogen, Carlsbad, CA, USA) which was diluted to 1:200 using the TNB buffer (100 mM Tris-HCl (pH 7.5), 150 mM NaCl, 0.5% Blocking reagent). The antibody solution (100 μL) was added to the slide at 37 °C for 30 min and then washed for 5 min thrice in TNT buffer (100 mM Tris-HCl (pH 7.5), 150 mM NaCl, 0.05% Tween 20) before being transferred to room temperature. A FITC-labeled goat anti-rabbit antibody (Jackson ImmunoResearch Laboratories, West Grove, PA, USA) was diluted to 1:250 using the TNB buffer. Then 100 uL of the antibody solution was added to the slide and incubated at 37 °C for 30 min, and was washed thrice in TNT buffer at room temperature for 5 min. The slide was immersed in 70%–100% ethanol series for dehydrating chromatin. Chromosomes were counterstained with 0.05 μg/mL of 4′,6-diamidino-2-phenylindole (DAPI) and mounted in 1,4-diazabicyclo[2.2.2]octane (DABCO) anti-fading solution (50% glycerol and 2% DABCO in PBS). All slides were observed on a Leica DM 2500 microscope (Leica Microsystems, Wetzlar, Germany) equipped with a CCD camera. FISHView EXPO 4.0 software (Applied Spectral Imaging, Migdal HaEmek, Israel) was used for image analysis.

### 3.5. Cytochrome b Gene Cloning and Analysis

DNA was extracted from 5 to 10 mg of muscle tissue of each of the five specimens of *O. barbatulum* and *O. alticorpus*. The complete Cyt *b* gene was amplified using the FishcytB-F primer 5′-ACCACCGTTGTTATTCAACTACAAGAAC-3′ and TruccytB-R primer 5′-CCGACTTCCGGATTACAAGACCG-3′ [30]. The reaction solution consisted of approximately 5 ng genomic DNA, 50 pmol each of the forward and reverse primers, 25 mM dNTP, 0.05–0.1 mM MgCl_2_, 10× buffer, and 5 U *Taq* polymerase (Takara Shuzo, Shiga, Japan), and brought up to 125 μL with Milli-Q water (Millipore, Billerica, MA, USA). The PCR program included one cycle of 4 min at 95 °C, 38 cycles of 1 min at 94 °C, 50 s at 54 °C, and 1 min at 72 °C, followed by a single further extension of 10 min at 72 °C. We evaluated 10 μL of each product on a 0.8% agarose gel to check PCR success and confirm product sizes. The remaining PCR products were run on 0.8% agarose gels and purified using a DNA Clean/Extraction kit (GeneMark, Taichung, Taiwan). Purified DNA was subcloned into the pGEM-T easy vector (Promega, Madison, WI, USA) and transformed into *Escherichia coli* JM109 strain. Plasmid DNA was isolated using a mini plasmid kit (Geneaid, Taichung, Taiwan). Different clones from each of five individuals of *O. barbatulum* and *O. alticorpus* were sequenced on an Applied Biosystems (ABI, Foster City, CA, USA) automated DNA sequencer ABI3730x1 using a Bigdye sequencing kit (Perkin-Elmer, Wellesley, MA, USA) as mentioned above.

In total, 10 Cyt *b* sequences were subcloned and deposited in GenBank. Homologous sequences of *O. ovale*, *O. lini*, *O. simum*, *Acrossocheilus cinctus*, *A. paradoxus*, *Varicorhinus maroccanus*, *V. beso*, *V. steindachneri*, *Barbaus barbus*, *B. rebeli*, *Spinibarbus hollandi*, and *S. denticulatus* derived from NCBI (Accession nos. JX074246, JQ343982, HM536801, JX066772, HQ443699, AF287457, JQ716388, AF180865, KC465927, AF090791, NC026129, and NC021616) were selected as reference sequences. All sequences were aligned using ClustalW [31] and then manually checked. Intraspecific and interspecific genetic distances and number of different nucleotides were calculated using MEGA software [29]. Variable site numbers, parsimony informative site numbers among the samples of the two *Onychostoma* species, and the intraspecies nucleotide diversity of *O. alticorpus* and *O. barbatulum* specimens were computed by DnaSP v5 [32]. The phylogenetic trees of Cyt *b* sequences were constructed using NJ methods [28]. Cluster confidence level was assessed using a bootstrap analysis with 1000 replications.

## 4. Conclusions

*Onychostoma barbatulum* and *O. alticorpus* have identical chromosome numbers 2*n* = 50, but variable karyotypes. The 5S rDNAs of these two *Onychostoma* species were cloned, sequenced, and mapped by FISH on metaphase plates. The 5S rDNA sequences showed differences in nucleotide lengths of NTRs with the chromosome location being pericentromeric in *O. barbatulum* and subtelomeric in *O. alticorpus*. The Cyt *b* gene sequences of *O. barbatulum* and *O. alticorpus* clearly revealed that these two species are not sister species and the *Onychostoma* genus is not monophyletic; nevertheless, their morphological similarities may be due to environmental adaptation.
